# Self-Reported Fractures in Dermatitis Herpetiformis Compared to Coeliac Disease

**DOI:** 10.3390/nu10030351

**Published:** 2018-03-14

**Authors:** Camilla Pasternack, Eriika Mansikka, Katri Kaukinen, Kaisa Hervonen, Timo Reunala, Pekka Collin, Heini Huhtala, Ville M. Mattila, Teea Salmi

**Affiliations:** 1 Coeliac Disease Research Center, Faculty of Medicine and Life Sciences, University of Tampere, 33014 Tampere, Finland; pasternack.m.camilla@student.uta.fi (C.P.); mansikka.eriika.k@student.uta.fi (E.M.); katri.kaukinen@staff.uta.fi (K.K.); kaisa.hervonen@staff.uta.fi (K.H.); timo.reunala@uta.fi (T.R.); 2 Department of Dermatology, Tampere University Hospital, 33521 Tampere, Finland; 3 Department of Internal Medicine, Tampere University Hospital, 33521 Tampere, Finland; 4 Department of Gastroenterology and Alimentary Tract Surgery, Tampere University Hospital, 33521 Tampere, Finland; pekka.collin@uta.fi; 5 Faculty of Social Sciences, University of Tampere, 33014 Tampere, Finland; heini.huhtala@staff.uta.fi; 6 Division of Orthopedics and Traumatology, Department of Trauma, Musculoskeletal Surgery and Rehabilitation, Tampere University Hospital, 33521 Tampere, Finland; ville.mattila@staff.uta.fi

**Keywords:** dermatitis herpetiformis, coeliac disease, fracture, bone health, quality of life

## Abstract

Dermatitis herpetiformis (DH) is a cutaneous manifestation of coeliac disease. Increased bone fracture risk is known to associate with coeliac disease, but this has been only scantly studied in DH. In this study, self-reported fractures and fracture-associated factors in DH were investigated and compared to coeliac disease. Altogether, 222 DH patients and 129 coeliac disease-suffering controls were enrolled in this study. The Disease Related Questionnaire and the Gastrointestinal Symptom Rating Scale and Psychological General Well-Being questionnaires were mailed to participants; 45 out of 222 (20%) DH patients and 35 out of 129 (27%) of the coeliac disease controls had experienced at least one fracture (*p* = 0.140). The cumulative lifetime fracture incidence did not differ between DH and coeliac disease patients, but the cumulative incidence of fractures after diagnosis was statistically significantly higher in females with coeliac disease compared to females with DH. The DH patients and the coeliac disease controls with fractures reported more severe reflux symptoms compared to those without, and they also more frequently used proton-pump inhibitor medication. To conclude, the self-reported lifetime bone fracture risk is equal for DH and coeliac disease. After diagnosis, females with coeliac disease have a higher fracture risk than females with DH.

## 1. Introduction

Coeliac disease is a systemic autoimmune disorder triggered by gluten and characterized by small-bowel mucosal villous atrophy. It has a highly heterogeneous clinical picture including intestinal, extraintestinal, and asymptomatic manifestations [1]. A number of comorbidities are associated with coeliac disease, one of which is metabolic bone disease predisposing to bone fractures [2]. At the time of diagnosis, coeliac disease patients frequently suffer from decreased bone mineral density (BMD) [2,3], which in turn may be a risk factor for fractures. Decreased BMD is not limited to only patients with severe gastrointestinal symptoms; it also occurs in subclinical and asymptomatic coeliac disease patients [4,5,6]. Once diagnosed, coeliac disease is treated with a life-long gluten-free diet (GFD). Strict adherence to a GFD typically improves bone health in coeliac disease, but full bone recovery is often not reached in adult coeliac disease patients [7]. Fracture risk in coeliac disease has been studied extensively, and based on a recent meta-analysis, it can be concluded that the risk of fractures in coeliac disease is increased by 30% for any fractures and by 69% for hip fractures [8]. 

Dermatitis herpetiformis (DH) is one of the well-established extraintestinal manifestations of coeliac disease [9]. In DH, dietary gluten induces an itchy, blistering rash, which responds to a GFD [10]. Since a GFD often alleviates the intensively itching rash fairly slowly, patients with severe symptoms are additionally treated with dapsone medication at the beginning of the dietary treatment to alleviate the skin symptoms more quickly [11]. At diagnosis, DH patients also suffer from coeliac disease-type small-bowel mucosal villous atrophy or inflammation. Occasionally gastrointestinal symptoms also occur, but they are often minor [9,12]. It seems presumable that the increased risk of bone fractures would also be associated with DH, but bone complications in DH have been studied scantily and the results are thus far conflicting [13,14,15,16,17]. The only study focusing on the fracture risk in DH found no increase in risk [13]. The aim of the current study was to discover whether DH patients have an increased bone fracture risk similar to the one known to exist in coeliac disease. A further objective was to study the factors associated with increased bone fracture risk in DH, and to assess the burden related to fractures. 

## 2. Materials and Methods 

### 2.1. Patients, Controls, and Study Design

All patients with DH within the catchment area of the city of Tampere are diagnosed and treated at a special outpatient clinic at Tampere University Hospital’s Department of Dermatology. The diagnosis of DH is based on clinical symptoms and the demonstration of granular immunoglobulin A deposits in perilesional skin biopsies studied with direct immunofluorescence [18]. From 1970 onwards, data have been prospectively collected from all patients diagnosed with DH. All adult DH patients alive in December 2015 and diagnosed before December 2014 (*n* = 413) were recruited to the study. The control group comprised 222 biopsy-proven coeliac disease patients diagnosed at Tampere University Hospital over the same time period who were suffering from abdominal symptoms at the time of diagnosis. 

Self-administered study questionnaires (see below for more detail) were mailed to the patients and controls. A second round of questionnaires were sent to all non-respondent patients and controls under 80 years old. For the DH patients, the total response rate was 56% (237 out of 413). Of these responders, 15 patients were excluded for having a coeliac disease diagnosis made more than one year prior to the DH diagnosis. The remaining 222 DH patients constituted the study cohort. For the coeliac disease controls, the final response rate was 59% (130 out of 222), and one patient was excluded because of a DH diagnosis. The patients’ medical records were reviewed and the clinical, serological, and histological severity of the disease and the use of dapsone were recorded. 

The DH patients and coeliac disease controls received a full written explanation of the aims of the study and they gave their written informed consent. The Regional Ethics Committee of Tampere University Hospital approved the study protocol. 

### 2.2. Questionnaires

Three self-administrated questionnaires were used in this study: the Disease Related Questionnaire (DRQ), which was specifically designed for this study, and the general Psychological General Well-Being (PGWB) and Gastrointestinal Symptom Rating Scale (GSRS) questionnaires, which have been widely used in coeliac disease studies. The DRQ includes both open questions and multiple-choice questions. Patients were asked to report all experienced bone fractures during their lifetime, the year of each fracture, and the type of trauma causing the fracture. The DRQ also includes questions about the respondent’s sociodemographic and lifestyle characteristics, presence of comorbidities, use of long-term medication, and current weight and height. In addition, the questionnaire also enquires about previous and current clinical symptoms related to coeliac disease and DH and the strictness of the respondent’s GFD. 

The PGWB is a 22-item questionnaire used to evaluate quality of life and well-being [19,20]. It covers six emotional states: anxiety, depressed mood, self-control, positive well-being, general health, and vitality. All of the items use a six-grade Likert scale, where value one represents the poorest and value six the best possible well-being. The total PGWB score thus ranges from 22 to 132 points, with a higher score indicating a better quality of life. 

The GSRS is a 15-item questionnaire used to assess the severity of five groups of gastrointestinal symptoms: diarrhoea, indigestion, constipation, abdominal pain, and reflux [21]. The questionnaire uses a seven-grade Likert scale for each item, one symbolizing no symptoms and seven indicating the most severe symptoms. The final scores are calculated as a mean for each sub-dimension and the total GSRS score as the mean of all 15 items. A higher score indicates more severe symptoms. 

### 2.3. Fractures

The self-reported fractures were categorized based on whether they had occurred before or after the DH or coeliac disease diagnosis. The traumas causing the fractures were evaluated, and if the trauma was considered sufficient to cause a bone fracture in any person (traffic accidents, high-energy sports fractures), the fracture was excluded from further analysis. Fractures diagnosed as stress fractures were also excluded from all further analysis. 

### 2.4. Statistical Analysis

Median values, minimum and maximum values, and interquartile ranges (IQR) were used to describe the continuous variables. All testing was two-sided and *p* < 0.05 was considered statistically significant. The chi-squared test was used in cross-tabulations and the Mann–Whitney *U* test was used for assessing changes between groups. Kaplan–Meier survival analysis was used to compare the cumulative incidence of fractures between the groups. The odds ratios (OR) and 95% confidence intervals (CI) were calculated by using binary logistic regression analysis. For fracture incidence rates, 95% CI were calculated assuming the number of fractures to have a Poisson distribution. All of the statistical analyses were performed with SPSS version 20 (IBM SPSS Statistics for Windows, Version 20.0. IBM Corp., Armonk, NY, USA) in cooperation with a statistician.

## 3. Results

In total, 101 of the 222 DH patients (45%) and 104 of the 129 coeliac disease controls (81%) were female. The DH patients were younger at the time of diagnosis than the coeliac disease controls (Table 1). At the time of the study, there were no differences in age or body mass index between the groups. The median follow-up time after diagnosis was 23 years (range 2–53) for the DH patients and 20 years (range 1–43) for the coeliac disease controls. The DH patients and the coeliac disease controls reported a total of 128 fractures, of which 9 excess-trauma fractures and 5 stress fractures were excluded from further analysis. There were no statistical differences between the groups in terms of the number of study participants who reported a fracture (Table 1). 

**Figure 1:**
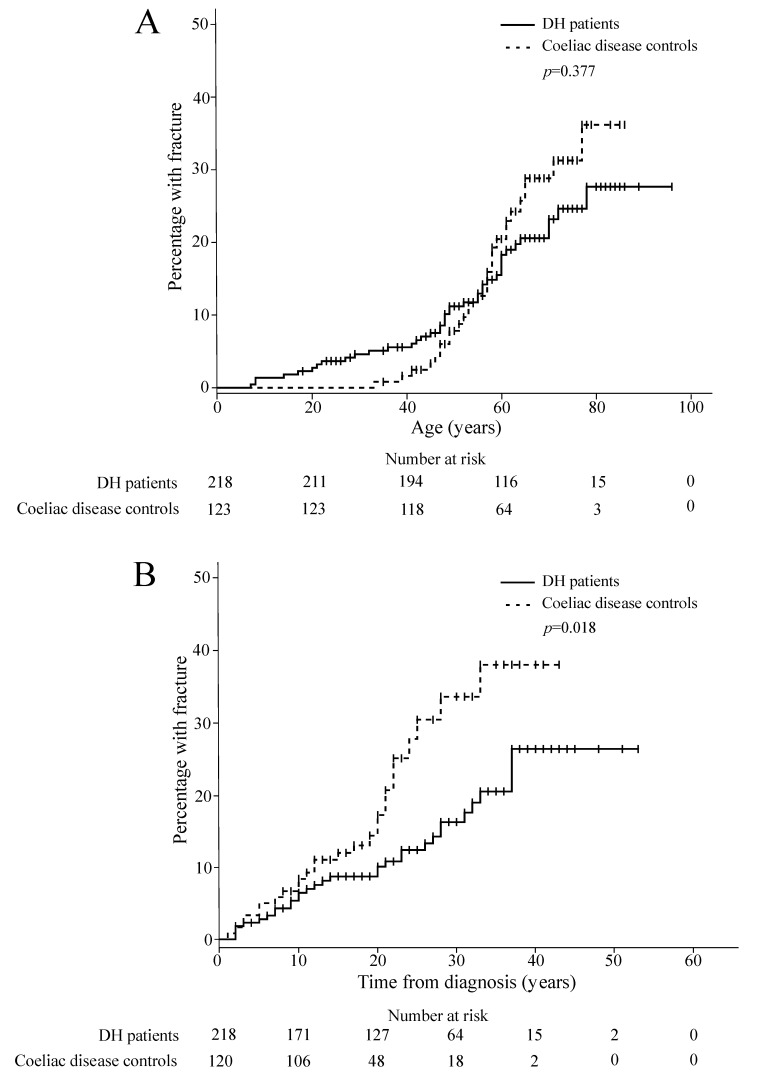
Kaplan–Meier cumulative incidence of the first fracture (**A**) and for first fracture after diagnosis (**B**) for the dermatitis herpetiformis (DH) patients and the coeliac disease controls.

The fracture incidence rate per 10^5^ person-years for the first fracture was 317 (95% CI 228–431) for the DH patients and 388 (95% CI 259–558) for the coeliac disease controls. For the first fracture after DH or coeliac disease diagnosis, the fracture incidence rates per 10^5^ person-years were 629 (95% CI 427–894) for the DH patients and 1083 (95% CI 707–1589) for the coeliac disease controls. In the binary logistic regression analysis, the risk of fracture for the coeliac disease group did not statistically significantly differ from the DH group before (OR 1.47, 95% CI 0.88–2.43, *p* = 0.141) or after adjustment for gender and age at the time of the study (adjusted OR 1.04, 95% CI 0.60–1.79, *p* = 0.891). In the Kaplan–Meier analysis, neither the cumulative lifetime fracture incidence (Figure 1A) nor the incidence before diagnosis (*p* = 0.127) significantly differed between the groups. The cumulative incidence of fractures after the diagnosis was statistically significantly higher in the coeliac disease group than in the DH group (Figure 1B). When the genders were analysed separately the difference was observed for female (*p* = 0.021) but not for male patients (*p* = 0.291). 

In both study groups, patients with fractures were more often female, had more often been diagnosed with osteoporosis, and more often had multiple long-term illnesses (Table 2). At the time of the study, those with fractures were more often using proton-pump inhibitors (PPI) and vitamin D and calcium supplementation than those with no reported fractures (Table 2). The current usage of hormone replacement therapy and diuretics was more common among the DH patients with reported fractures than those without, but this was not seen in the coeliac disease controls. There were no differences in the smoking habits or current adherence to a GFD, the amount of weekly exercise, or the use of glucocorticoids or bisphosphonates between those with and without fractures in either groups (Table 2). Compared to coeliac disease controls with fractures, DH patients with fractures were statistically significantly more often male, and they were younger at the time of the diagnosis (Table 2) and at the time of the first fracture (median 48 years vs. 57 years, *p* = 0.048). 

The DH patients with fractures more frequently had severe villous atrophy in the small bowel at the time of the diagnosis than those without fractures, but the difference in the histological severity of the disease did not reach statistical difference (Table 3). There were no differences in the duration of skin symptoms before DH diagnosis, the severity of skin symptoms, or the presence of gastrointestinal symptoms at diagnosis between DH patients with fractures and those without, but DH patients with fractures had used dapsone medication statistically significantly longer after diagnosis than those without fractures (Table 3). 

The severity of the gastrointestinal symptoms as measured with the GSRS total score did not differ between DH patients or coeliac disease controls with and without fractures (Table A1). However, DH patients with fractures reported higher GSRS reflux subscores than those without fractures (median 1.5 vs. 1.0, *p* = 0.012). This was also seen in the coeliac disease controls, although without reaching statistical significance (median 1.5 vs. 1.0, *p* = 0.083). The quality of life measured with the PGWB questionnaire was decreased in DH patients with fractures compared to those without in total score (median 106 vs. 112, *p* = 0.006) and in all other subscores except depression (Table A1). This same phenomenon was not observed in the coeliac disease controls (Table A1). 

## 4. Discussion

In the current study, the lifetime fracture risk in DH was found not to differ from that in coeliac disease, which is well known to be linked to increased bone fracture risk [8]. However, it was found that females with coeliac disease had more fractures after coeliac disease was diagnosed than females with DH after DH diagnosis. The sole previous study addressing bone fracture risk in DH, with a respectable 846 DH patients from the United Kingdom, found no increased fracture risk in DH compared to the general population (hazard ratio 1.1) [13]. However, the study had limitations, as their observation period was rather short (median 3.7 years, 3496 person-years) and the data collected regarding adherence to GFD treatment was scanty. In the current study, the fracture incidence was smaller than in the work by Lewis et al., but the results are not comparable, as different study methods and follow-up times were used. In addition, our study groups had good adherence to a GFD, which is likely to decrease the fracture incidence. Other than the study by Lewis et al., bone health in DH has been investigated in four small studies focusing on the DH patients’ BMD. Two studies found that DH patients have a decreased BMD compared to non-DH controls but better BMD than coeliac disease controls [14,15], and two studies found that the BMD in DH patients did not differ from that expected [16,17]. 

It is acknowledged that strict adherence to a GFD increases BMD in coeliac disease patients [2,7], and thus probably decreases the risk of bone fractures in the long term. In the current study, there were no differences in the strictness of GFD at the time of the study in the DH patients and the coeliac disease controls with and without fractures (Table 2). Overall, the GFD in this study cohort was strict, showing that bone fractures also tend to occur when the patients adhere to the diet well. However, we do not have short-term data about the strictness of the diet after the diagnosis. The duration of dapsone use after diagnosis was longer for DH patients reporting fractures compared to those who did not (Table 3). Longer requirement of dapsone usage suggests more active and prolonged rash, which might be a consequence of ongoing gluten consumption from dietary lapses on GFD. Less strict GFD after diagnosis in turn would be a logical cause for increased risk of fractures.

Bone deterioration in coeliac disease is considered a consequence of autoimmune reaction. The autoimmune reaction causes local and systemic chronic inflammation, which in turn causes micronutrient deficiency and activates a network of cytokines that have deleterious consequences for bone remodeling [22]. As DH and coeliac disease principally share the same pathogenetic mechanisms, it is accurate to assume that the same mechanisms explain the increased fracture risk also observed in DH. However, our study showed that after diagnosis, the female coeliac disease controls experienced more fractures than the female DH patients. This difference could be caused by the on-going small-bowel inflammation that remains in coeliac disease even after the recovery of the mucosal architecture [23]. In addition, the age at diagnosis was higher for the coeliac disease controls compared to the DH patients. The higher age at diagnosis has been linked to less complete bone recovery following GFD, and it seems that the ability to bone recovery is the least satisfactory for peri- and post-menopausal women [3,24]. The higher age at diagnosis might also indicate that coeliac disease patients have suffered from the untreated disease longer than DH patients, and untreated disease particularly before puberty would have unfavorable effects on bone health.

The prevalence of gastrointestinal symptoms at the time of diagnosis was not linked to reported fractures, nor was any symptom other than reflux in the GSRS questionnaire at the time of the study. Consistently with more severe reflux symptoms detected, both the DH patients and the coeliac disease controls with fractures reported using PPI as a long-term medication more often than those without fractures. The usage of PPI medication has been reported to increase the risk of fractures at any site in a recent meta-analysis [25]. Thus, caution with PPI medications should be advised, although based on the experienced reflux symptoms, there is a clear need for PPI medication in certain individuals with DH and coeliac disease. 

This study showed that the DH patients who reported fractures had a decreased quality of life compared to those with no fractures. Although we do not know if this decrease in life quality is caused by the burden of fractures or simply explained by a higher rate of multiple long-term illnesses detected in patients with fractures, we think that more attention should be directed to fractures in DH. In this study cohort, very few of the DH patients with fractures had been diagnosed with osteoporosis, which suggests that BMD measurements may not have been carried out systematically in the presence of fractures. This limits the awareness and motivation to adequately treat patients suffering from low BMD, and thus it also limits the prevention of additional fractures. 

The strengths of this study are the large cohorts of patients with biopsy-proven DH and coeliac disease with strict inclusion and exclusion criteria, and thus a minimal possibility for misclassification bias. The cohorts were diagnosed and treated by specialists during the same time period, and they included patients with different severities of the disease from the same geographic area. The limitations of this study should be recognized, however. The fractures in this study were self-reported, which is not ideal. Nevertheless, questionnaire studies have in fact proven to be rather reliable [26], and although we know that the results are underestimations of the true occurrence due to recall bias [27], the groups in this study are comparable because they were both studied in a similar manner. Another limitation is that we have not taken the site of the fracture into account in our analysis, so we do not know how this would have differed between the groups. The study also compared only the fractures between DH and coeliac disease, and we did not enrol healthy controls. The gender distributions between our cohorts were unequal, but they correspond to the true distributions in both diseases and therefore the cohorts cannot be considered unrepresentative. 

## 5. Conclusions

In conclusion, the fracture risk in DH is analogous to that in coeliac disease, which is a disease widely documented to be associated with increased bone fracture risk. However, after the diagnosis, the fracture risk was higher in coeliac disease than in DH for female patients. The severity of the skin disease did not correlate with the fracture risk in DH, but the reflux symptom and usage of PPI medication were linked to an increased fracture risk. The quality of life was shown to be decreased in DH patients with a history of fractures, which indicates that more attention should be directed to the fracture risk in DH, and BMD measurements should not be overlooked.

## Figures and Tables

**Table 1:** Demographic data and reported fractures among 222 dermatitis herpetiformis (DH) patients and the 129 coeliac disease controls.

	DH Patients (*n* = 222)		Coeliac Disease Controls (*n* = 129)		*p*-Value
	*n*	%	*n*	%	
Female	101	45	104	81	<0.001
Age at diagnosis, median (range), years	37 (5–78)		42 (7–72)		0.027
Age at the time of the study, median (range), years	65 (18–96)		66 (35–86)		0.654
BMI ^1^ at the time of the study, median (range), kg/m^2^	26 (17–40)		26 (15–46)		0.714
Reported fractures	45	20	35	27	0.140
Before diagnosis	13	6	3	3	0.143
After diagnosis	31	14	26	22	0.080
Reported multiple fractures	15	7	12	9	0.388

^1^ BMI, Body mass index.

**Table 2:** Demographic data, strictness of gluten-free diet (GFD), and clinical data for the dermatitis herpetiformis (DH) patients and the coeliac disease controls with and without fractures.

	DH Patients (*n* = 222)		*p*-Value	Coeliac Disease Controls (*n* = 129)		*p*-Value
	With Fracture (*n* = 45)	Without Fracture (*n* = 177)		With Fracture (*n* = 35)	Without Fracture (*n* = 94)	
Female, %	58 *	42	0.064	97	74	0.002
Age at diagnosis, median (range), years	34 (7–78) *	37 (5–78)	0.652	45 (23–59)	40 (7–72)	0.428
Age at the time of the study, median (range), years	68 (22–85)	65 (18–96)	0.343	68 (51–82)	63 (35–86)	0.020
Smoking at the time of the study, %			0.581			0.218
Non-smoker	69	68		66	70	
Ex-smoker	17	22		31	20	
Current smoker	14	10		3	10	
Exercise at the time of the study, %			0.341			0.396
Not at all	11	11		6	10	
1 to 3 times per week	55	43		57	44	
4 to 7 times per week	34	46		37	46	
Dietary adherence to GFD at the time of the study, %			0.858			0.646
Strict ^1^	70	73		80	86	
Dietary lapses less than once a month	21	18		17	10	
Dietary lapses more than once a month	7	8		3	3	
Normal diet	2	1		0	1	
Diagnosed with osteoporosis, %	11 *	2	0.024	40	11	0.001
Multiple long-term illnesses ^2^, %	33	19	0.033	43	26	0.057
Use of long-term medication at the time of the study, %						
Proton-pump inhibitor	21	9	0.029	26	11	0.034
Hormone replacement therapy	14	4	0.008	3	11	0.155
Any glucocorticoid medication	18	12	0.349	26	16	0.205
Vitamin D and calcium supplementation	39	10	<0.001	43	25	0.045
Bisphosphonates	5	3	0.545	9	6	0.506
Diuretics	21	9	0.028	11	8	0.491

^1^ No dietary lapses, ^2^ Two or more of the following diseases: thyroid disease, diabetes, hypercholesterolaemia, hypertension, rheumatoid disease, coronary artery disease. * *p* < 0.05 when the DH patients with fractures were compared to the coeliac disease patients with fractures.

**Table 3:** Disease-related characteristics presented as percentages, median values, and interquartile ranges (IQR) for the dermatitis herpetiformis (DH) patients with fractures and those without fractures.

	DH Patients		*p*-Value
	With Fracture (*n* = 45)	Without Fracture (*n* = 177)	
Year of DH diagnosis, median (IQR)	1990 (1976–2000)	1991 (1982–2002)	0.076
Duration of skin symptoms prior to DH diagnosis, median (IQR), months	12 (6–60)	10 (5–24)	0.183
Severity of skin symptom at diagnosis, %			0.818
Mild	19	15	
Moderate	46	50	
Severe	35	35	
Presence of gastrointestinal symptoms at the time of diagnosis, %	47	49	0.886
Small-bowel histology at diagnosis, %			0.405
Normal	16	24	
PVA ^1^	35	39	
SVA/TVA ^2^	49	37	
Use of dapsone after diagnosis, %	79	77	0.854
Duration of dapsone, median (IQR), months	60 (12–171)	24 (12–60)	0.031

^1^ PVA, Partial villous atrophy; ^2^ SVA/TVA, Subtotal or total villous atrophy.

## Supplementary Materials

Supplementary File 1

## Appendix A

**Table A1:** Median values and interquartile ranges for the Psychological General Well-Being (PGWB) and Gastrointestinal Symptoms Rating Scale (GSRS) totals and subscores for the dermatitis herpetiformis (DH) patients and the coeliac disease controls with and without fractures. In the PGWB, a higher score indicates a better quality of life, and in the GSRS, a higher score indicates more severe symptoms.

	DH Patients (*n* = 222)			Coeliac Disease Controls (*n* = 129)		
	With Fracture (*n* = 45)	Without Fracture (*n* = 177)	*p*-Value	With Fracture (*n* = 35)	Without Fracture (*n* = 94)	*p*-Value
GSRS						
Total	1.9 (1.3–2.3)	1.6 (1.3–2.1)	0.191	1.9 (1.5–2.5)	1.7 (1.4–2.5)	0.472
Diarrhoea	1.7 (1.0–2.3)	1.3 (1.0–2.0)	0.116	2.0 (1.0–2.7)	1.7 (1.0–2.5)	0.635
Indigestion	2.0 (1.3–2.5) *	1.8 (1.5–2.5)	0.630	2.4 (1.8–2.8)	2.0 (1.5–3.0)	0.343
Constipation	1.3 (1.0–2.3)	1.3 (1.0–2.3)	0.568	1.7 (1.3–2.5)	1.7 (1.0–2.7)	0.572
Pain	1.7 (1.2–2.3)	1.3 (1.0–2.0)	0.130	1.7 (1.3–2.3)	1.7 (1.3–2.3)	0.517
Reflux	1.5 (1.0–2.0)	1.0 (1.0–1.5)	0.012	1.5 (1.0–2.5)	1.0 (1.0–2.0)	0.083
PGWB						
Total	106 (94–113)	112 (101–119)	0.006	110 (93–120)	106 (97–116)	0.629
Anxiety	25 (22–27)	26 (23–28)	0.020	27 (21–29)	25 (23–28)	0.757
Depression	17 (16–18)	18 (16–18)	0.311	17 (15–18)	17 (15–18)	0.948
Well-being	17 (15–19)	18 (16–20)	0.007	18 (15–19)	17 (16–20)	0.941
Self control	16 (15–17)	16 (15–17)	0.052	16 (13–17)	16 (14–17)	0.888
General health	13 (11–15)	15 (13–16	0.012	13 (11–16)	13 (11–15)	0.712
Vitality	18 (17–21)	20 (17–21)	0.029	19 (17–20)	18 (16–20)	0.665

* *p* < 0.05 when the DH patients with fractures were compared to the coeliac disease patients with fractures.
