# Supplementary material for: Self-Reported Fractures in Dermatitis Herpetiformis Compared to Coeliac Disease
Source: Nutrients. 2018 Mar 14;10(3):351. doi: 10.3390/nu10030351 (PMC5872769; doi:10.3390/nu10030351)
Supplement: Supplementary File 1 [file nutrients-10-00351-s001.pdf]

**Supplementary table 1.** Median values and interquartile ranges for the Psychological General Well-Being (PGWB) and Gastrointestinal Symptoms Rating Scale (GSRS) totals and subscores for the dermatitis herpetiformis (DH) patients and the coeliac disease controls with and without fractures. In the PGWB, a higher score indicates a better quality of life, and in the GSRS, a higher score indicates more severe symptoms.

| | **DH patients (n=222)** | | | **Coeliac disease controls (n=129)** | | |
|---|---|---|---|---|---|---|
| | **With fracture (n=45)** | **Without fracture (n=177)** | ***p*-value** | **With fracture (n=35)** | **Without fracture (n=94)** | ***p*-value** |
| GSRS | | | | | | |
| Total | 1.9 (1.3–2.3) | 1.6 (1.3–2.1) | 0.191 | 1.9 (1.5–2.5) | 1.7 (1.4–2.5) | 0.472 |
| Diarrhoea | 1.7 (1.0–2.3) | 1.3 (1.0–2.0) | 0.116 | 2.0 (1.0–2.7) | 1.7 (1.0–2.5) | 0.635 |
| Indigestion | 2.0 (1.3–2.5)* | 1.8 (1.5–2.5) | 0.630 | 2.4 (1.8–2.8) | 2.0 (1.5–3.0) | 0.343 |
| Constipation | 1.3 (1.0–2.3) | 1.3 (1.0–2.3) | 0.568 | 1.7 (1.3–2.5) | 1.7 (1.0–2.7) | 0.572 |
| Pain | 1.7 (1.2–2.3) | 1.3 (1.0–2.0) | 0.130 | 1.7 (1.3–2.3) | 1.7 (1.3–2.3) | 0.517 |
| Reflux | 1.5 (1.0–2.0) | 1.0 (1.0–1.5) | 0.012 | 1.5 (1.0–2.5) | 1.0 (1.0–2.0) | 0.083 |
| PGWB | | | | | | |
| Total | 106 (94–113) | 112 (101–119) | 0.006 | 110 (93–120) | 106 (97–116) | 0.629 |
| Anxiety | 25 (22–27) | 26 (23–28) | 0.020 | 27 (21–29) | 25 (23–28) | 0.757 |
| Depression | 17 (16–18) | 18 (16–18) | 0.311 | 17 (15–18) | 17 (15–18) | 0.948 |
| Well-being | 17 (15–19) | 18 (16–20) | 0.007 | 18 (15–19) | 17 (16–20) | 0.941 |
| Self control | 16 (15–17) | 16 (15–17) | 0.052 | 16 (13–17) | 16 (14–17) | 0.888 |
| General health | 13 (11–15) | 15 (13–16 | 0.012 | 13 (11–16) | 13 (11–15) | 0.712 |
| Vitality | 18 (17–21) | 20 (17–21) | 0.029 | 19 (17–20) | 18 (16–20) | 0.665 |

*$p < 0.05$ when the DH patients with fractures were compared to the coeliac disease patients with fractures
